# Adipose tissue-derived mesenchymal stem cells and chitosan/poly (vinyl alcohol) nanofibrous scaffolds for cartilage tissue engineering

**DOI:** 10.1186/s13619-020-00045-5

**Published:** 2020-06-10

**Authors:** Ghada Nour-Eldeen, Mazen Abdel-Rasheed, Amira M. EL-Rafei, Osama Azmy, Gehan T. El-Bassyouni

**Affiliations:** 1 Molecular Genetics and Enzymology Department, National Research Centre, Dokki, Cairo, 12622 Egypt; 2 Stem Cell Research group, Medical Research Centre of Excellence, National Research Centre, Cairo, 12622 Egypt; 3 Reproductive Health Research Department, National Research Centre, 33 El-Buhouth St, Dokki, Cairo, 12622 Egypt; 4 Refractories, Ceramics and Building Materials Department, National Research Centre, Dokki, Cairo, 12622 Egypt

**Keywords:** Adipose tissue, Mesenchymal stem cells, Nanofibrous scaffolds, Osteoarthritis, Cartilage tissue engineering

## Abstract

Osteoarthritis (OA) has been defined as a chronic inflammatory joint disease characterized by progressive articular cartilage degeneration. Recently growing interest in regenerative medicine, using cell therapy and tissue engineering, where cellular components in combination with engineered scaffolds and bioactive materials were used to induce functional tissue regeneration. In the present study, nanofibrous scaffold based on chitosan (CS)/poly (vinyl alcohol) (PVA) were used to develop biologically functionalized biomaterial to mimic the extracellular matrix, allowing the human adipose tissue derived mesenchymal stem cells (ADSCs) to proliferate and differentiate to chondrogenic cells. The morphology of the nanofibrous mat was examined using field emission scanning electron microscope (FE/SEM). The characteristic functional groups and the nature of the chemical bonds between atoms were evaluated using Fourier transform infrared spectroscopy (FTIR) spectrum. Characterization of the seeded cells was morphologically evaluated by scanning electron microscopy and by flow cytometry for the expression of the stem cell surface markers. The differentiation potential was verified after chondrogenic induction by analyzing the expression of chondrogenic marker genes using real-time (RT PCR). Current study suggest significant potential for the use of ADSCs with the nanofibrous scaffolds in improving the osteoarthritis pathology.

## Background

Osteoarthritis is known to be chronic, debilitating joint disease. It is characterized by progressive articular cartilage degeneration, subchondral bone remolding, and marginal reactive new bone formation causing pain and stiffness during movement. About 9.6% of males and 18% of females above 60 years old have symptomatic osteoarthritis all over the world (Refugees UNHC for Refworld n.d.; Kaur et al. 2018). WHO “World Health Organization” determined that worldwide over 150 million people complain of symptoms of osteoarthritis (Liu et al. 2018). Currently, classical treatment for osteoarthritis is directed toward symptomatic treatment, specifically pain treatment, with no ability to boost regeneration of damaged cartilages and to prevent further degenerative processes.

As a consequence, substantial health and socio-economic burden globally (Wallace et al. 2017). Autologous chondrocyte implantation (ACI) as well as matrix-induced autologous chondrocyte implantation (MACI), gave incredible guarantee 80% of patients demonstrating great outcomes at 10 years (Bentley et al. 2013). However, complications may occur such as graft failure, periosteal hypertrophy and delamination (Wood et al. 2006; Peterson et al. 2000). Additionally, it has been accounted for that cells may lose their phenotype during expansion (Benya and Shaffer 1982; Takata et al. 2011). Consequently, growing interest in regenerative medicine, using cell therapy, where cells were directly injected into the blood or into tissues, and tissue engineering, where cellular components in combination with engineered scaffolds and bioactive materials were used to induce functional tissue regeneration.

Mesenchymal stem cells (MSCs) have the characteristics of attachment to plastic culture vessels and facility to express CD44, CD73, CD90, and CD105 but not CD45, CD34, and CD14 cell surface markers. MSCs are multipotent cells, having both hypo-immunogenic and immunomodulatory characteristics, which let them capable to home damaged tissues and pledge healing via repair processes. They are believed to possess low immunogenicity, as they express low levels of major histocompatibility complex (Huang et al. 2016; Molina et al. 2015; Xu and Li 2014). It has recently been suggested that MSCs have a new line for management of osteoarthritis in accordance with their capability of differentiation into chondrocytes, and the paracrine effects of secreted bioactive substances that might be more important than differentiated cells in enhancing repair responses (Beris et al. 2005), through the anti-inflammatory and immunomodulatory effects of MSCs that may delay the development of osteoarthritis (Counsel et al. 2015).

The designing of scaffold to have composition, biological, mechanical and physiochemical properties that imitate extra cellular matrix (ECM) of the damaged tissue is considered one of the significant tools for tissue engineering (Yang et al. 2007). Scaffolds should non-immunogenic, non-toxic, biocompatible and biodegradable. In addition, scaffold should have suitable surface properties that provide in-vitro cell adhesion, ingrowth and provide necessary space for neo-vascularization in-vivo (Salgado et al. 2004). To target specific tissue engineering applications, many natural and synthetic polymers have been investigated.

Chitosan (CS) is a naturally derived biodegradable polysaccharide commonly used in tissue engineering because of its biodegradable, biocompatible, and non-toxic properties. Therefore, it is a safe material for use in biomedical applications (Ibrahim et al. 2016; Ismail et al. 2018). CS is a compound of glucosamine and *N*-acetyl glucosamine linked in β (1–4) manner. CS, as a derivative of chitin, has as intrinsic antibacterial activity. Therefore, it can decrease the infection rate of experimentally induced osteomyelitis by *Staphylococcus aureus* in rabbits. It has been mentioned that CS with a variety of delivery materials such as alginate, hydroxyapatite, hyaluronic acid, and growth factors have a potential application in orthopedic tissue engineering (Li et al. 2005; Yamane et al. 2005; Hsieh et al. 2005).

Interestingly, it has been reported that CS blended with poly (vinyl alcohol) (PVA) have good mechanical and chemical characteristics (Charernsriwilaiwat et al. 2010). PVA is a water-soluble synthetic resin that produced via polymerization of vinyl acetate monomer. PVA was used in controlled release systems and due to its biocompatible nature; it has a variety of biomedical uses (Soppimath et al. 2000).

Water-soluble polymers including polysaccharides (such as alginate) as well as synthetic polymers such as [Poly (ethylene oxide), PEO], [Poly (vinyl alcohol), PVA], [Poly (vinyl pyrrolidine, PVP] are known to be more biocompatible than other organic-soluble polymers. The electrospinning process which of relatively low cost and low toxicity, is an interesting approach for regenerative medicine requirements (Jimmy and Kandasubramanian 2020; Krishnan et al. 2013).

There is another important factor in tissue engineering which is the scaffold fabrication method. Recently researcher focused on the electrospinning for the manufacture of nanofibrous scaffolds that are suitable for the 3D cell cultures for tissue regeneration (Li et al. 2002). Continuous nanofibers in electrospinning are formed due to the electrostatic Coulombic repulsive forces applied throughout elongation of the viscoelastic solution as it strengthens to form a fiber. Electrospinning is a simple method to produce nanofibers that is similar to the collagen part of the extracellular matrix (ECM). Fibers produced by this method have the features of large surface-to-volume ratio, and high porosity that are needed for tissue engineering, by which nanofibers allow better cellular spreading, attachment and supply efficient nutrient to the cells (Hezma et al. 2017; El-Rafei 2015; El-Rafei et al. 2017).

The aim of the current study was to establish suitable physiologically and biochemically relevant microenvironment allowing ADSCs proliferation and differentiation into chondrocyte-like cells using CS/PVA nanofiber scaffolds.

## Methods

### Preparation of CS/PVA solutions

Various combinations of the factors that control the quality of the electrospun fibers (e.g., composition of the electrospinning solution and its viscosity, applied voltage, and distance between collector and nozzle) were investigated by try-and-error method. The reported conditions are the optimal ones that gave fibers a homogeneous structure and high quality. Fibers were prepared by the dissolution of chitosan (medium molecular weight, deacetylated chitin, poly (D-glucosamine), Aldrich) in 2% acetic acid solution for 2–3 h at room temperature until the formation of a clear solution. PVA (typical molecular weight = 124,000, 87–89% hydrolyzed, Sigma-Aldrich) was gradually added to the chitosan solution at 75 ± 5 °C while stirring for an additional 2 h in order to enhance the dissolution of the PVA crystals. After complete dissolution, the prepared solution was stirred overnight in a magnetic stirrer at room temperature to obtain homogeneous solution. The CS/PVA nanofibrous mat was prepared using electrospinning apparatus (NaBond Company, China). The solution was transferred into a 10 ml plastic syringe equipped with a metallic capillary nozzle connected to a high power supply. The voltage was adjusted at 25 kV. The inner diameter of the used nozzle was 0.49 mm and its height from the collector was set at 10 cm. The selected flow rate was 0.7 mL/h. The electrospun fibers were collected on an aluminum foil collector. Then, the electrospun mat was collected, dried for 24 h then stored for further characterization.

### Characterization of the CS/PVA

The microstructure of as-spun nano fibers was examined using Field Emission Scanning Electron Microscopy (FE-SEM) (Philips XL30, Netherlands).The Fourier transform infrared spectroscopy (FTIR) spectrum of nanofibrous mats was recorded using a Vertex 70 spectrometer (Bruker Optiks, Germany). The nanofibers were mixed with KBr powder, at a weight ratio of 1/100 nanofiber/KBr, then pressed to form a disc. The spectrum was in the spectral range of 4000-400 cm^− 1^ with spectral resolution of 2.0 cm^− 1^ and scan speed of 2 mm^− 1^. The viscosity of the blend solutions CS/PVA was measured using a rotating Viscometer (Brookfield viscometer DV-E, USA).

### Isolation of adipose tissue Mesenchymal stem cells (ADSCs)

ADSCs were obtained from freshly isolated subcutaneous fat from healthy donors (*n* = 5, age: 22–41) undergoing cesarean section surgery as described previously (Gimble and Guilak 2003), after written informed consent. The study was approved by the ethics committee of National Research Centre (NRC). Adipose tissue was minced, washed 3 times with phosphate-buffered saline (PBS). Subsequently, adipose tissue pieces were digested while shaking by 1 mg/mL collagenase type I (Gibco, USA) for 2 h at 37 °C. The released cells and residual adipose tissue were centrifuged at 1200 rpm for 10 min. The pellet was re-suspended in a complete culture medium of alpha-DMEM (Gibco, USA) with 10% fetal bovine serum (FBS), 100 U/mL Penicillin and 100 μg/mL Streptomycin. Cells were seeded at a density of 1 × 10^5^ cells/mL in 25 mm tissue flasks and incubated in a humidified atmosphere of 5% CO_2_ at 37 °C. Sub-cultured when cells reached 80% confluent (Alstrup et al. 2019).

### Characterization of ADSCs

The undifferentiated ADSCs were analyzed for Mesenchymal stem cell surface markers using flow cytometry, multi-lineage differentiation potential, and stemness gene markers expression. The cells were characterized with regard to a set of markers characteristic for ADSCs including CD73, CD105, CD90, CD271, CD34 and HLA-DR (Bayati et al. 2013; Zahran et al. 2012). ADSCs differentiation to osteocytes, adipocytes, and chondrocytes were induced using StemPro™ induction media, for 21 days. The differentiation were detected by using alizarin red stain, oil red stain, and Alcain blue stain respectively. The total RNA was extracted followed by cDNA synthesis. The following primers were used for the evaluation of Oct- 4, Sox-2, Nanog, Oct-4 fwd: 50-GCGAAGCAGGAGTCGGGGT-30; Oct-4 rev: 50-AGCGGCAGATGGTCGTTTG-30; Sox-2 fwd: 50-ACACCAATCCCATCCACACT-30; Sox-2 rev: 50-GCAAACTTCCTGCAAAGCTC-30; Nanog fwd: 50-CCTATGCCTGTGATTTG-30; Nanog rev: 50-AGAAGTGGGTTGTTTGC-30. The PCR cycle parameters were as follows: 94 °C for 5 min, 94 °C for 30 s, 58–62 °C for 30 s and 72 °C for 45 s (30 cycles),then a final extension for 10 min at 72 °C. The PCR products were analyzed by 2% agarose gel stained with ethidium bromide.

### Cell seeding of scaffolds and culture

CS/PVA scaffolds were sterilized by UV light for 1 h before cell seeding. The cell seeding of scaffolds was performed in a 6 well plates at density of 1 × 10^5^ cells/well. Human Adipose-derived Stem Cells (hADSCs) were harvested from the cell culture plates with 0.05% trypsin. The cell-seeded scaffolds were cultured in alpha-DMEM supplemented with 10% FBS and 1% penicillin-streptomycin.

### Cell viability and proliferation assay

Cell viability on the scaffolds and tissue culture plate was assessed by MTT cell proliferation assay kit (Roche Applied Science, Penzburg, Germany), following manufacturer instructions. After 1, 7, and 14 days of cell culture, 20 μl MTT reagent was added to each well of the microtiter plates containing the scaffolds, and cells were incubated for 4 h at 37 °C. After 200 μl solubilization solution (DMSO; Roche Diagnostics, Indianapolis, IN, USA) was added to each well, the plates were incubated overnight. The absorbance was measured at 595 nm using a microplate reader (Bio-Rad Laboratories, Inc., Hercules, CA, USA).

### Cell adhesion assay

Cells were seeded on both scaffold, and micro tire culture plate as a control surface at density of 1 × 10^4^ cells/well and same surface area. Cells were allowed to adhere for 4, 16, and 24 h. The non-adherent cells were washed gently with PBS and adherent cells in both conditions were counted.

### Apoptosis assay

Cellular apoptosis was analyzed using an Annexin V-EGFP/PI kit (Nanjing KeyGEN Biotech. Nanjing, China). Briefly, cell pellets were re-suspended in a binding buffer followed by incubation with 5 ml of Annexin V (conjugated with FITC) and propidium iodide (PI) staining in a dark place for 10 min. Fluorescence was analyzed by the FACS Caliber Flow Cytometer. Cells positive for Annexin V-FITC and negative for PI were considered apoptotic and those positive for both Annexin V-FITC and PI were deliberated necrotic.

### Chondrogenic differentiation

Cell seeded-scaffolds were cultured in StemPro™ Chondrogenesis Differentiation media (Gibco, USA) for 21 days, and the media were changed twice a week.

### Scanning Electron microscopy (SEM)

For both differentiated and undifferentiated groups, cell attachment and differentiation were evaluated via SEM. The cell-seeded scaffolds were washed twice with PBS and fixed with 2.5% glutaraldehyde for 30 min followed by 2% osmium tetroxide treatment for 30 min. After washing steps, the scaffolds were dehydrated in a series of ethanol solutions with increasing concentrations (30% to 100%) and finally dried with hexamethyl disilazane (HDMS). For SEM analysis, the cell-seeded scaffolds were sputter coated with a 5 nm gold layer. A silver/carbon sputter coating was applied to the examined samples. The scaffolds were examined with a Quanta 400F scanning electron microscope (FEI Company, Oregon, USA) (Liu et al. 2013).

### Reverse transcription quantitative polymerase chain reaction (RT-qPCR)

Total RNA was isolated from human MSCs, using TRIzol. cDNA synthesis and real-time PCR were performed as described previously (Vimalraj and Selvamurugan 2015). cDNA was amplified in a 20-μl reaction mixture containing FastStart SYBR Green Master (Roche Applied Science, Penzberg, Germany) and a specific primer pair of each cDNA according to the published sequences. PCRs were prepared in duplicate and heated to 95 °C for 10 min followed by 40 cycles of denaturation at 95 °C for 15 s, annealing at 60 °C for 1 min, and extension at 72 °C for 20 s with a specific primer pair of each cDNA following by sequences. RNA expression levels were quantified by means of a Light Cycler 480 (Roche Diagnostics, Mannheim, Germany) in relation to glyceraldehyde-3-phosphate dehydrogenase (GAPDH) housekeeping gene. The primer sets were as follows: **COL2A1:** GAGACAGCATGACGCCGAG (forward) and GCGGATGCTCTCAATCTGGT (reverse), **aggrecan:** TCGAGGACAGCGAGGCC (forward) and TCGAGGGTGTAGCGTGTAGAGA (reverse), **SOX-9:** GTACCCGCACTTGCACAAC (forward) GTAATCCGGGTGGTCCTTCT (reverse), **MMP13:** AACGCCAGACAAATGTGACC (forward) and AGGTCATGAGAAGGGTGCTC (reverse).

### Statistical analysis

T-test was used to evaluate the statistical significance of the results. Differences with *P* values < 0.05 were considered significant. The cycle threshold (Ct) values for each sample were given automatically by the I Cycler according to the amplification curves. The baseline and threshold values were manually set as recommended by the PCR array user manual. The selected threshold was 20.0 and the baseline cycles 2–10. The relative mRNA expression was calculated using the ΔΔCt (delta delta cycle threshold) method (Vimalraj et al. 2014), and the data were normalized, across all plates, to the following housekeeping GAPDH**.**

## Results

### Microstructure of electrospun mat

Various Blended weight ratios of CS/PVA solutions, were used to control the quality of the electrospun fibers following the try-and-error method. The optimal composition of chitosan /PVA was chosen depending on the produced nanofibers morphology.

**Fig. 1:**
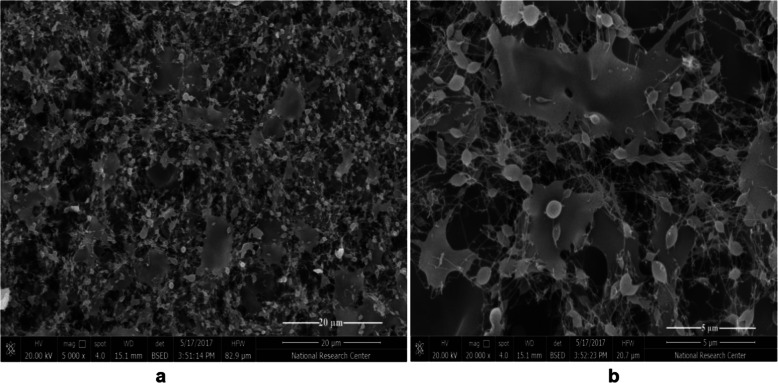
SEM images of the as-spun mate of CS/ PVA with weight ratio = 20:80 **a** Low magnification (5000 x), **b** High magnification (20,000 x)

In case of spinning of blended solutions of CS/PVA with weight ratios more than 10:90; drops was not able to eject and consequently, blocked the needle. This might be owing to the high viscosity of the concentrated solution, which cause clogging of the needle. The viscosity of the solutions of compositions greater than CS/PVA =20:80 was too high to be spun. In the composition CS/PVA =20/80, the outcome electrospun fibers start to appear on the collector, however, a non-uniform ejection of the jet as shown in Fig. 1. This can be attributed to the viscoelastic force of the polymeric solution that lead to thinning of the charged jet less likely to happen. Consequently, the jet segment was prevented from being stretched by the constant Coulombic repulsion force. There was a suitable range of viscosity of the polymer solution within which the polymer solutions are electrospinable and beyond which droplets were likely to happen.

**Fig. 2:**
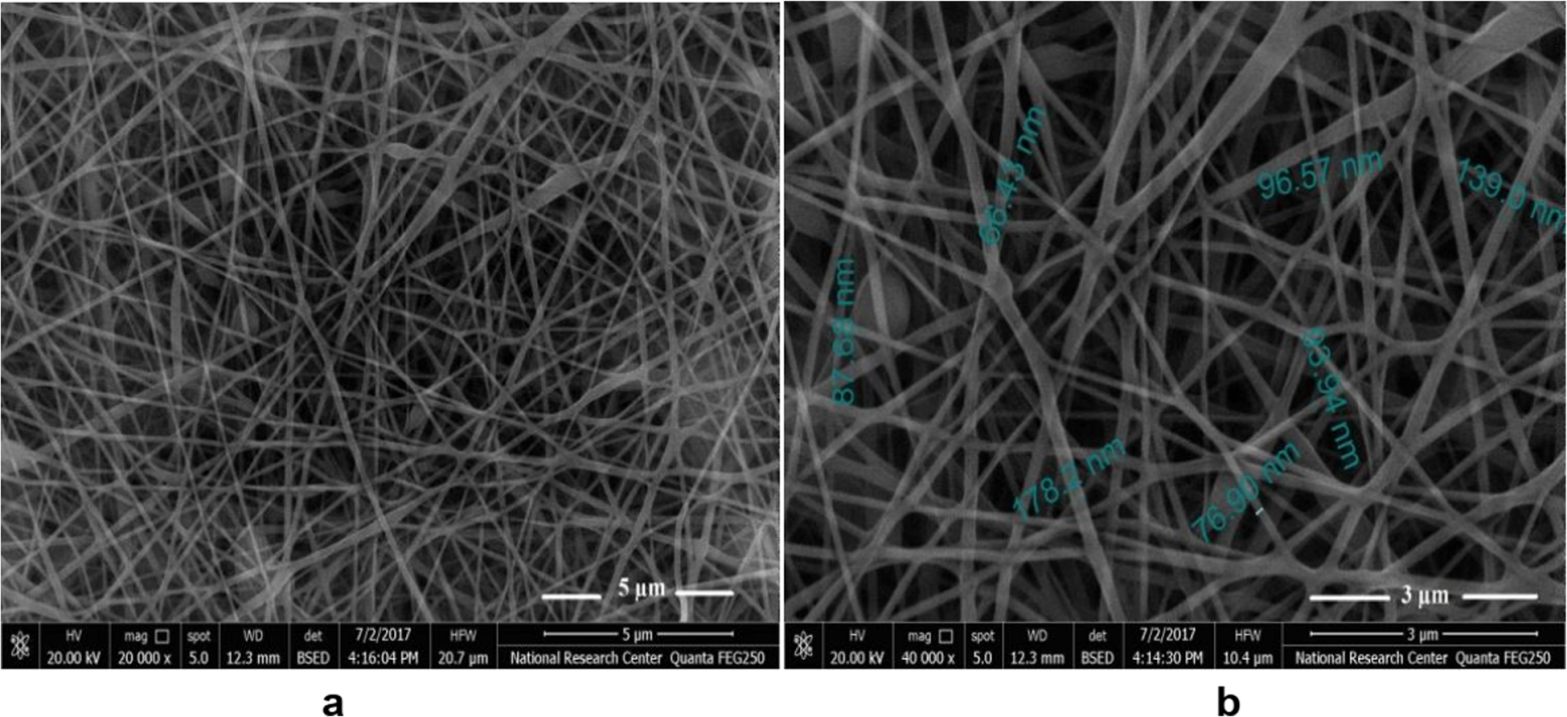
SEM images of the as-spun mat of CS/ PVA with weight ratios equal to 10:90 **a** at low magnification (20,000 x), **b** High magnification (40,000 x)

The suitable weight ratio was 10:90 for CS/PVA yield nanofiber microstructures mats with homogenous architecture mats as presented in Fig. 2. The following electrospinning parameters were applied: electrical potential of 25 kV, TCP: 10 and flow rate 0.7 mL/h and the viscosity value of the solution was almost 1195 cP. The diameter of the nanofibers was within 50–200 nm and the beads clearly disappeared.

### FTIR of the Nanofibers

**Fig. 3:**
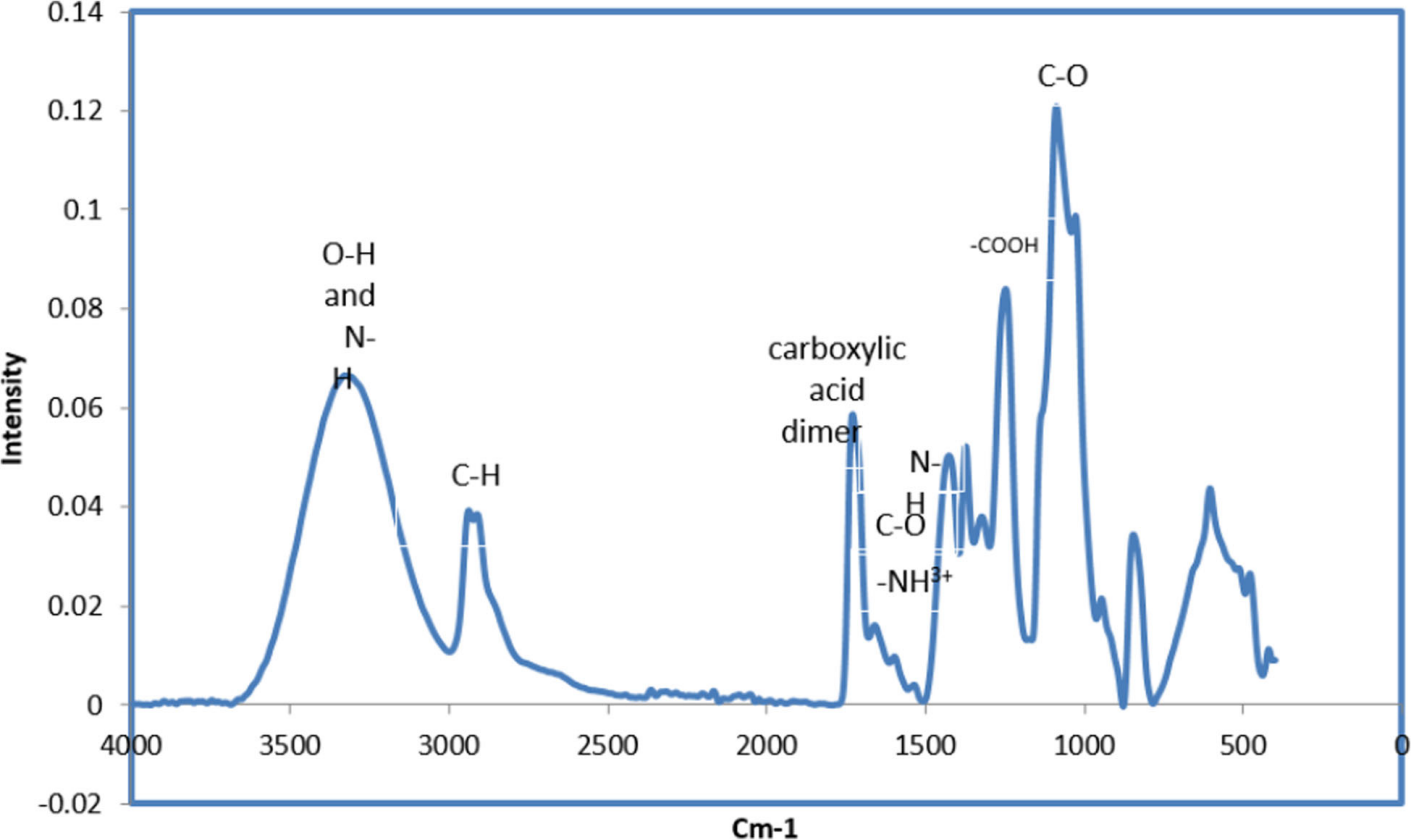
FTIR spectrum of the electrospun CS/PVA nanofiber mat

Carboxylic acid [−COOH] and symmetric deformation of amino [−NH^3+^] groups, arises from the two peaks at 1427 cm^− 1^ and 1537 cm^− 1^ due to the ionization of the primary amino groups in the acidic medium, respectively as presented in the FTIR spectrum of the CS/PVA nanofibers mat shown in Fig. 3**.** The peak at 1730 cm^− 1^ was ascribed to the carboxylic acid dimer (Alhosseini et al. 2012). This peak is due to the acetic acid employed for dissolving the chitosan. The peak positioned at 1248 cm^− 1^ related to the C-O of the CH_2_OH chitosan group forming a hydrogen bond with the OH group of the PVA, confirming the fabrication of CS/PVA blend nanofibers (Gholipour et al. 2009). The absorption peak at 1661 cm^-1^ was attributed to the C-O stretching of the acetyl group (amide I), as well as the C=C stretching vibration of the PVA. The band at 1599 cm^-1^ was assigned to the N-H bending and stretching amide II. The C-O asymmetric stretching band, was around 1089 cm^− 1^. The broad absorption peak; in the range 3600–3000 cm^− 1^, could be related to the O-H and N-H stretching vibrations (Jia et al. 2007).

### Characterization of isolated hADMSCs

**Fig. 4:**
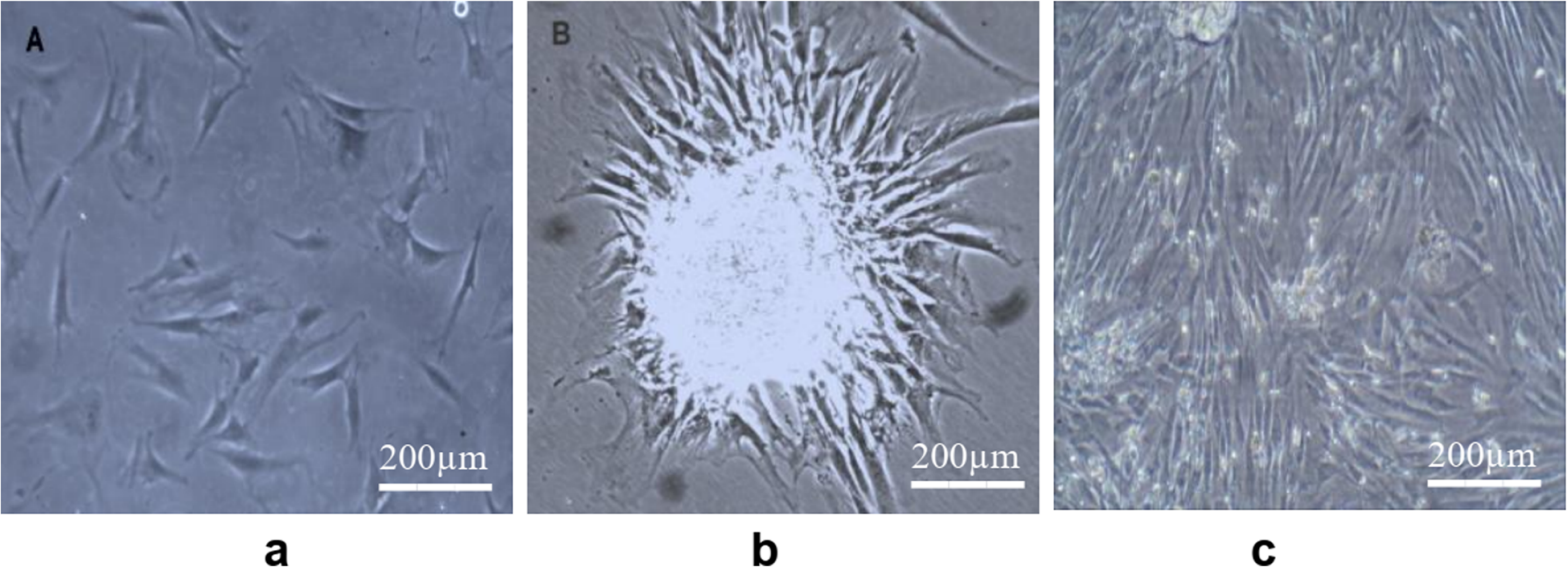
Morphological characterization of isolated ADSCs, Scale Bar 200 μm. **a** Representative image of spindle-shaped ADSCs, **b** Colony forming unit. **c** 90% confluent at 10th day

**Fig. 5:**
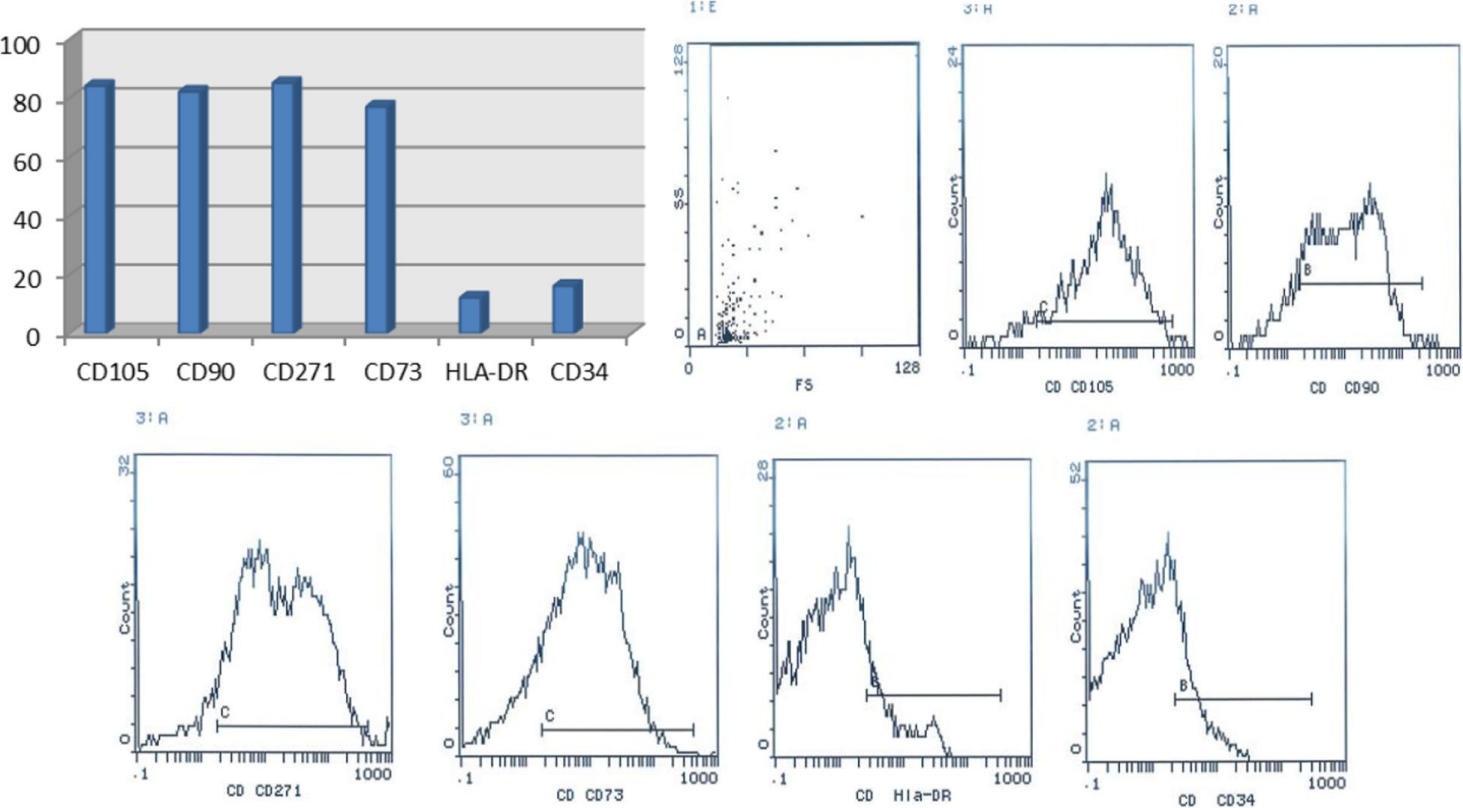
Immunophenotypic expression of mesenchymal stem cell surface marker with flow cytometry analysis of ADSCs showing that cells were positive for (CD73, CD105, CD90, and CD271) and were negative for (CD34, and HLA-DR)

**Fig. 6:**
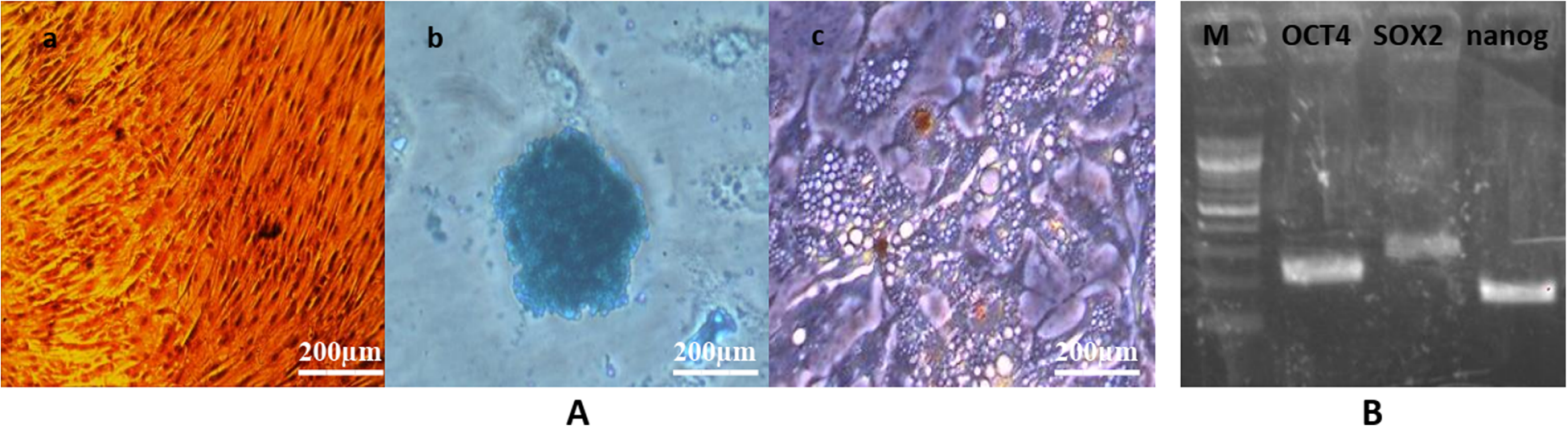
(**A**) In vitro multi-linage differentiation of human adipose tissue derived mesenchymal stem cells (ADSCs): osteogenic differentiation demonstrated by alizarin red stain in the form of dark orange mineral deposits (a), chondrogenic differentiation was stained positive for glucosaminoglycans by Alcain blue stain (b), specific oil red stain indicated adipogenesis induced lipid droplets observed in red colour (c). Scale bars: 200 μm. (**B**) reverse transcriptase (RT-PCR) determined ADSCs expression of pluripotent markers Nanog, Oct-4 and Sox-2

Fibroblast-like cells started to develop from the adipose tissue cell pellets between the 7th and 10th day of primary culture and reached up to 80–90% confluence in a whirlpool or radiating manner after 10 days **(**Fig. 4**)**. Cells were characterized as strongly positive for mesenchymal cell surface markers CD105 (endoglin, expression over 84%), CD90 (Thy-1, thymocyte antigen- 1, expression of more than 82% of cells), CD271 (LNGFR: low affinity nerve growth factor receptor, expression of more than 85% of cells), and CD73 (ecto-5′-nucleotidase, expression of more than 77% of cells) and low expression for HLA-DR (expression was less than 12%) and CD34 (early hematopoietic stem cell marker expression was less than 16%). Results confirm that the cultured cells presented the typical MSC immunophenotype: CD105+/CD73+/CD90+/CD271+/CD34−/HLA-DR− (Fig. 5**).** RT-PCR results demonstrated that ADSCs express the pluripotent markers Oct-4, Nanog and Sox-2 at passage 3 (Fig. 6). Differentiation potential was demonstrated at day 21 after induction through Calcium mineralization, which was confirmed by positive Alizarin Red S staining for osteogenesis, accumulation of lipid vacuoles for adipogenesis by oil red stain, and micromass pellet formation for chondrogenesis by Alcain blue stain.

### Cell adhesion and viability

**Fig. 7:**
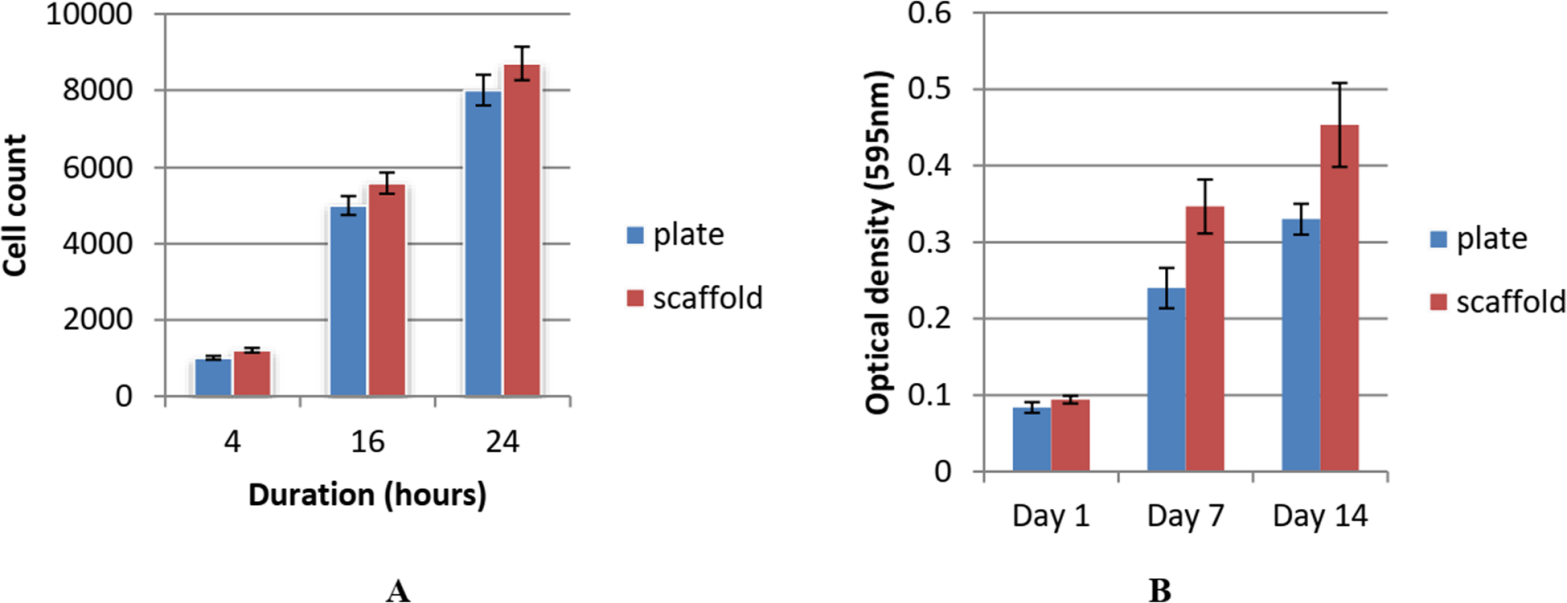
showing cell viability, proliferation and adhesion. **a** Cell adhesion assay. **b** MTT assay results using mean and standard deviation

Adhesion capability of seeded cells was almost the same on scaffold and plastic tissue culture plates as it was estimated for the same period and surface area (Fig. 7a). Statistically significant difference of viable cells at day 7 and day 14 of culture between plate and scaffold-seeded cells (*p*-value < 0.05) demonstrated by MTT assay (Fig. 7b).

### Apoptosis assay

**Fig. 8:**
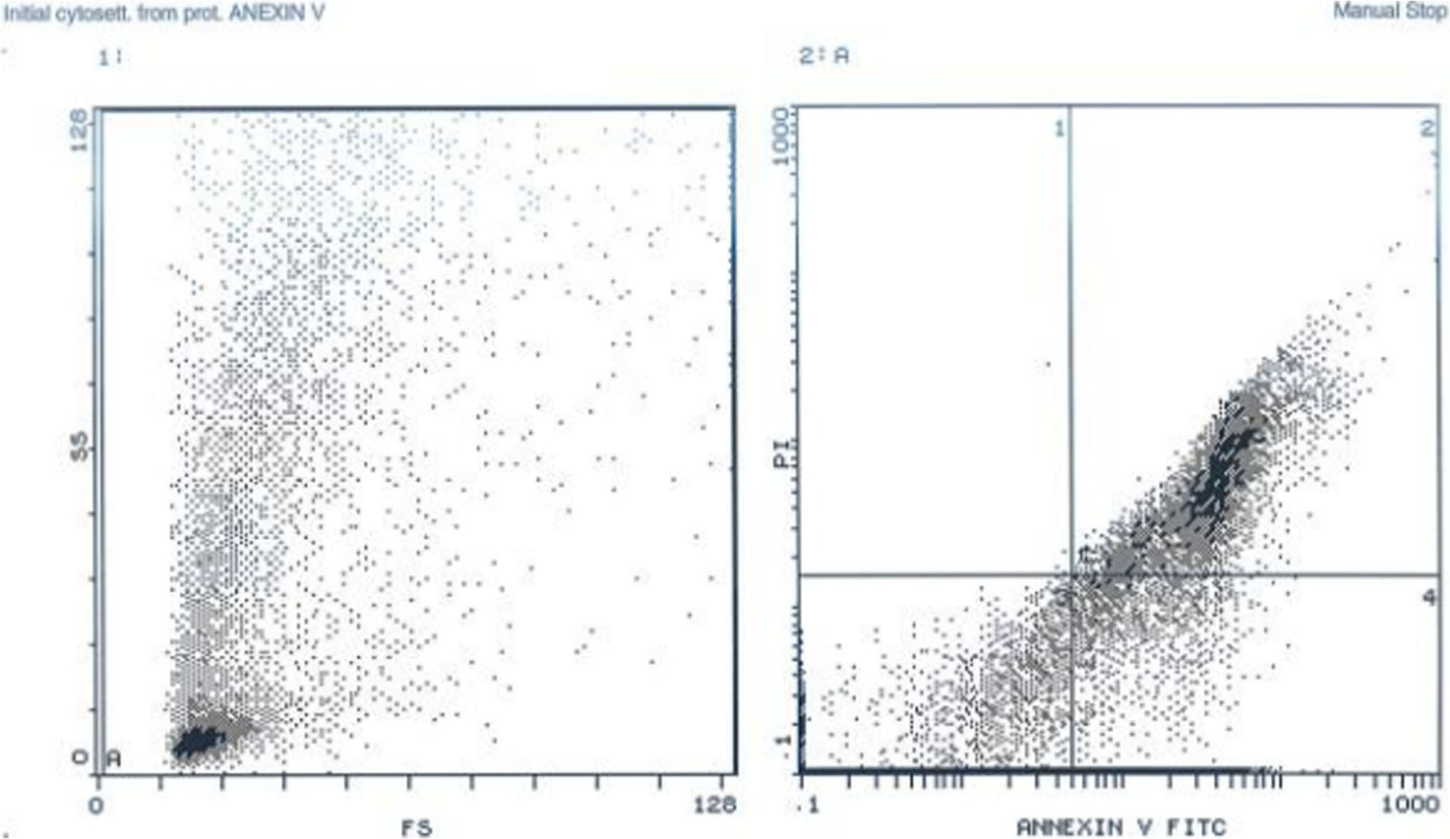
Apoptosis assay analyzed by Annexin V

Cells, which were positive for Annexin V-FITC and negative for PI, were considered apoptotic; while those cells, which were positive for both Annexin V-FITC and PI, were considered necrotic, as showed in (Fig. 8**)**, most of cells were living cells (more than 75%).

### Cell attachment and differentiation potential

**Fig. 9:**
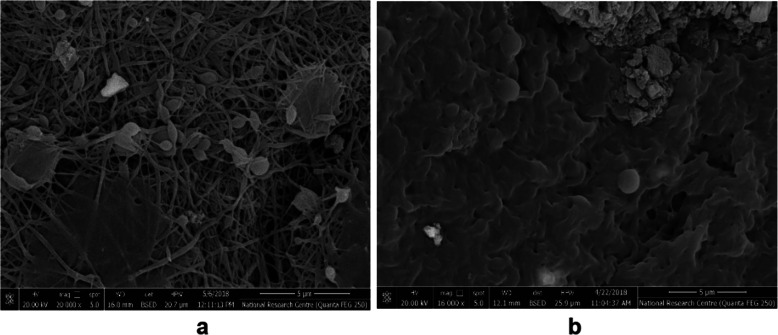
SEM images cells seeded on CS/PVA scaffold after 21 days, **a** spindle-shaped undifferentiated ADSCs, **b** Chondrocyte-like cells with spherical shape after culture in chondrogenesis differentiation medium

SEM images at day 21 determined, both undifferentiated ADSCs and ADSCs with chondrogenic differentiation media, cultured on scaffolds were distributed regularly forming a cell monolayer on top of the constructs, and filled the pore spaces (Fig. 9).

### Expression of Chondrogenic marker genes

**Fig. 10:**
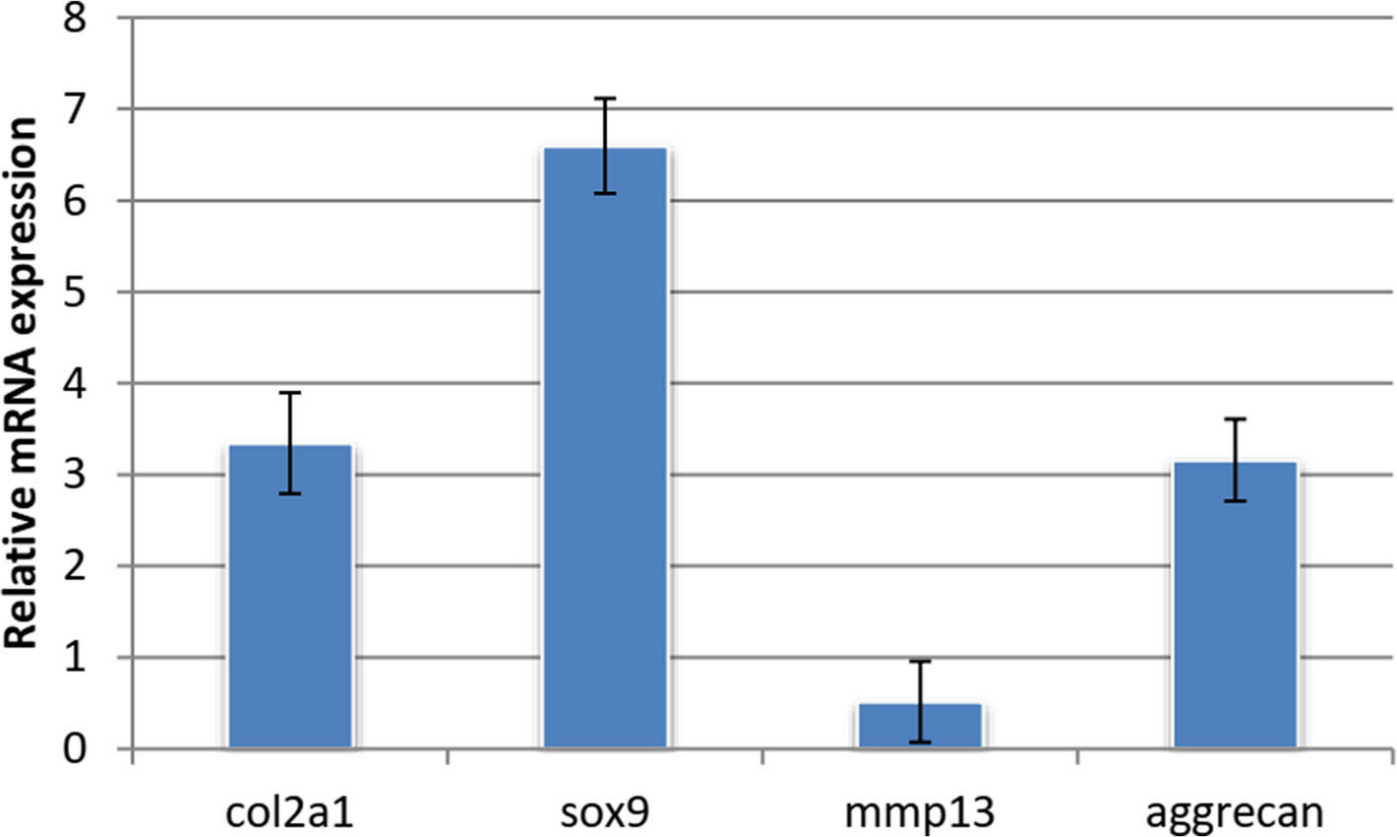
The mRNA expression levels of pluripotent stem cell markers were analyzed by real-time PCR and normalized to their respective GAPDH levels. Values represented as mean + SD

Quantitatively, the differentiation potential of ADMSC on the CS/PVA scaffolds was verified after chondrogenic induction by analyzing the expression of chondrogenic marker genes using real-time (RT PCR). In the present study, we analyzed expression of involved genes using real-time RT PCR. Expression of *COL2A1,*[a fibrillar collagen present in the cartilage and the vitreous humor of the eye] SOX9 [identifies the sequence CCTTGAG along with other members of the HMG-box class DNA-binding proteins, it acts through chondrocyte differentiation and, with steroidogenic factor 1] and Aggrecan, genes [the most abundant proteoglycan in cartilage, that during early development makes up much of the skeleton] were differentially upregulated, although MMP13 [convoluted in the breakdown of extracellular matrix in normal physiological processes] was downregulated (Fig. 10).

## Discussion

Articular cartilage is a hyaline tissue, but without any blood, lymphatic or nerve supply. It is characterized by a single cell type, chondrocytes, which are responsible for the synthesis of highly hydrated extracellular matrix. It is composed of collagen fibers, which provide tensile strength, and proteoglycan aggregates, mainly aggrecan (responsible for the compressive strength, attached along a filament of hyaluronic acid). Cartilage provides protection to the subchondral bone. It is considered as a lubricant and a shock absorber (Baugé and Boumédiene 2015; Pujol et al. 2008). During life, acute trauma, that may be happen, can cause articular cartilage defects. In addition, biochemical changes by aging may induce the degradation of cartilage matrix and result in chronic diseases such as osteoarthritis. Recently, osteoarthritis has been defined as progressive chronic inflammatory joint disease due to abnormal immune response, as early inflammatory response was induced by innate immune cells, while the chronic, relapsing course of inflammation of osteoarthritis was developed by adaptive immunity (Kandahari et al. 2015; Jaime et al. 2017).

Nanofiber scaffolds composed of ultra-fine biodegradable polymeric fibers morphologically similar to natural ECM have been widely emerged as potential scaffolds for cartilage tissue engineering. It merits referencing that while nanofibrous structures could mimic similar fiber diameters, composition, and alignment of the ECM of articular cartilage, the synchronization of these scaffolds with appropriate cells will accomplish the best tissue engineering outcomes for articular cartilage (Chiang and Jiang 2009). It was reported that MSCs affect joint microenvironment and facilitate tissue repair not only by their differentiation potential, but also via paracrine signals, which facilitate cartilage repair and decline the degenerative process of osteoarthritis.

The aim of this study is to measure the extent of biocompatibility of fabricated CS/PVA nanofibrous scaffolds by electrospinning to mimic the biological and biochemical milieu of the native (ECM) to encourage the isolated adipose tissue derived mesenchymal stem cells (ADSCs) proliferation and chondrogenic differentiation. In current study, non-seeded CS/PVA nanofibrous scaffold provided a significant high surface area to volume ratio as it consisted of long nanofibers with large surface area, small sized pore and big pore volume. Cells used for the study was at third passage of the 2D culture system as the differentiation potential of ADSCs decline with extended passaging (González-Cruz and Darling 2013).

Isolated ADSCs had typical spindle-shaped morphology with colony formation as seen in Fig. 4. Cells expressing certain immunophenotyic surface markers (CD105+ /CD73+ /CD90+ /CD271 + /CD34− /HLA-DR−) shown in Fig. 5, pluripotency markers (OCT4, SOX2, and Nanog) and also had multidifferentiation potential detected by Alizarin red stain for osteoblasts, Alcain blue stain for chondrocytes, and oil red stain for adipocytes as shown in Fig. 6, which was consistent with the International Society for Cellular Therapy (Krampera et al. 2013), as there is three criteria must be fulfilled for the MSC phenotype: adherence to plastic with characteristic morphology, appropriate immunophenotypic profile, and expression of multipotent differentiation potential. Cell viability was assessed by Annexin V as it showed 75% of cells were viable.

MTT test was used to determine the viability and proliferation of cells on days 1, 7, and 14 of being in culture on the scaffolds. As it is shown in Fig. 7, scaffold did induce any negative impact or toxic effect on the proliferation level of seeded cells. It was reported that MSCs had a weak attachment and proliferation at the first 3 days of culture. This delay, which is a constant biological procedure, may be due to the need of cellular adaptation with the new matrix and environment, as there is an alternation in the nutrient consistency or due to the presence of solvent remnant in media during the first few days (Kheradmandi et al. 2016).

SEM images also showed a uniform cell distribution and high scaffold biocompatibility and demonstrated good interaction between the cells and scaffold as a hopeful solution for tissue engineering. For chondrogenic differentiation of ADSCs on the CS/PVA nanofiber mat, ready to use chondrogenesis supplements for MSCs, StemPro A10071–01 chondrogenesis differentiation kits “Gibco/Life Technologies, Darmstadt, Germany” for 21 days. Detection methods for chondrogenic differentiation vary from lineage-specific immunological or histological assays to the direct detection of chondrocyte specific extracellular matrix (ECM) protein and gene expression.

The expression of both parathyroid hormone 1 receptor (Pth1r) and Indian hedgehog (Ihh) is land mark of maturation stage into prehypertrophic chondrocytes. Early hypertrophic chondrocytes express later collagen, type X, α1 (Col10a1) accompanied loss of Sox5, Sox6, Sox9, Col2a1 and Acan expression. Late hypertrophic chondrocytes, finally express only vascular endothelial growth factor A (VEGFA), matrix metalloproteinase 13 (Mmp13) and secreted phosphoprotein 1 (also known as, osteopontin/bone sialoprotein 1; Spp1). Expression of VEGFA and Mmp13 means that presence of endothelial cells, osteoclasts and osteoblast precursors of the growth plate.

Chondrogenic gene markers, which are known to be expressed at different stages of chondrogenic differentiation over time (Xu et al. 2008), including SOX9, Aggrecan and COL2A1, and MMP13 were estimated 21 days after induction by qRT-PCR. Expression of Sox-9 mRNA, which is the principal transcription factor for chondrogenic genes (Akiyama and Lefebvre 2011), was upregulated on CS/PVA scaffolds following chondrogenic stimulation. SOX9 required for chondrogenic lineage commitment by mesenchymal stem cell condensation (Quintana 2009). It was also reported that SOX9 inhibits the osteoblastogenesis key transcription factor runt-related transcription factor 2 (RUNX2) (Studer et al. 2012). Aggrecan as well as COL2A1 were significantly upregulated suggesting promotive impact of CS/PVA on chondrogenic differentiation of ADSCs in vitro. MMP-13, matrix metalloproteinase 13, was significantly down regulated.

Several studies reported that the process of endochondral ossification takes place during the progression from the early commitment to the late hypertrophic stage of chondrogenic differentiation (Gawlitta et al. 2010), This late hypertrophic progression initiates the formation of a mineralized cartilaginous matrix which is eventually converted into bone (Kronenberg 2003). It was reported that in vitro chondrogenically differentiated bone marrow-derived MSC express several hypertrophy-related genes, such as matrix metalloproteinase 13 (MMP-13) and type X collagen (Coleman et al. 2013), leading to unwanted formation of calcified matrix implanted subsequently in the sub cutis of nude mice (Dickhut et al. 2009).

## Conclusion

Adult mesenchymal stem cells along with biomaterial scaffolds seem to be attractive candidates for regenerating articular cartilage dysfunction, due to chondrogenic differentiation potential, and immunomodulatory characteristics. Current study suggest significant potential applications for human adipose tissue derived mesenchymal stem cells (ADSCs) with Chitosan/poly (vinyl alcohol) nanofibrous scaffolds in improving osteoarthritis pathology. Our future plan is to establish controlled animal model to study if human mesenchymal stem cells along with CS/PVA nanofibrous scaffolds can induce structural joint improvement for osteoarthritis invivo.

## Data Availability

Please contact author for data requests.
